# Perceived barriers and facilitators of using dietary modification for CKD prevention among African Americans of low socioeconomic status: a qualitative study

**DOI:** 10.1186/1471-2369-15-194

**Published:** 2014-12-06

**Authors:** Amber E Johnson, L Ebony Boulware, Cheryl AM Anderson, Tatpong Chit-ua-aree, Kimberly Kahan, LaPricia Lewis Boyér, Yang Liu, Deidra C Crews

**Affiliations:** Department of Medicine, Johns Hopkins University School of Medicine, Baltimore, Maryland USA; Welch Center for Prevention, Epidemiology and Clinical Research, Johns Hopkins Medical Institutions, Baltimore, Maryland USA; Department of Epidemiology, Johns Hopkins Bloomberg School of Public Health, Baltimore, Maryland USA; Division of General Internal Medicine, Department of Medicine, Duke University, Durham, North Carolina USA; Department of Family and Preventive Medicine, University of California San Diego, La Jolla, California USA; Division of Nephrology, Department of Medicine, Johns Hopkins University School of Medicine, Baltimore, Maryland USA; Division of Nephrology, Johns Hopkins University School of Medicine, Johns Hopkins Bayview Medical Center, 301 Mason F. Lord Drive, Suite 2500, Baltimore, MD 21224 USA

**Keywords:** Renal, Diet, Disparities, Race, Chronic kidney disease

## Abstract

**Background:**

Factors influencing the use of dietary interventions for modification of CKD risk among African Americans have not been well-explored. We assessed perceived barriers and facilitators of CKD prevention through dietary modifications among African Americans with low socioeconomic status (SES) and at high risk for CKD.

**Methods:**

We conducted a qualitative study involving three 90 minute focus groups of low SES (limited education, unemployed, uninsured, or income < $25,000/year) African American residents of Baltimore, Maryland (N = 17), who were aged 18-60 years, with no known history of CKD and (1) a family history of end stage renal disease and (2) self-reported diabetes, hypertension, cardiovascular disease, HIV or obesity. A trained moderator asked a series of 21 closed and open-ended questions. Group sessions were recorded, transcribed, and two independent investigators reviewed transcripts to identify common themes.

**Results:**

Participants’ mean (SD) age was 39.8 (12.4) years. Most (59%) were female and earned < $5,000/year (71%). One quarter (24%) had self-reported diabetes and over half had hypertension (53%). Few (12%) perceived their CKD risk as high. Perceived barriers to CKD prevention through dietary change included the expense and unavailability of healthy foods, family member preferences, convenience of unhealthy foods, and inability to break lifelong habits. They identified vouchers for healthy foods, family-based interventions, nutritional counseling and group gatherings for persons interested in making dietary changes as acceptable facilitators of dietary CKD prevention efforts.

**Conclusions:**

Low SES African Americans at high risk for CKD had limited perception of their risk but they identified multiple barriers and potential facilitators of CKD prevention via dietary modifications which can inform future studies and public health interventions.

**Electronic supplementary material:**

The online version of this article (doi:10.1186/1471-2369-15-194) contains supplementary material, which is available to authorized users.

## Background

African Americans are more than twice as likely to live in poverty as are whites [1] and socioeconomic factors are believed to contribute in complex ways to racial disparities in chronic kidney disease (CKD) [2]. Dietary factors may play a role in these disparities. Specific dietary patterns or components have been associated with greater risk of prevalent [3, 4] or incident CKD [5, 6]. Several dietary patterns have been associated with CKD progression in observational studies [7–10], including diets which are non-adherent to the Dietary Approaches to Stop Hypertension (DASH) trial diet [11]. Additionally, diets high in fruits and vegetables and low in dietary acid load (similar to the DASH diet [12]) have demonstrated kidney-related benefits in controlled studies [13, 14]. African Americans are more likely to experience food insecurity [15] and/or live in “food deserts” [16], which may contribute to documented racial disparities in diet quality [17]. For example, African Americans with hypertension are less likely to follow a DASH trial accordant diet than are whites [18], despite their being shown to potentially receive the greatest blood pressure benefit from the DASH diet [19].

Although reasons for poorer diet quality among African Americans compared to whites are not fully understood, they are likely multifactorial, including African Americans’ perceptions of healthful dietary practices, access to healthful foods, cultural and familial norms, and preferences [16, 20–22]. Prior studies have demonstrated African Americans’ perceived risk of CKD is low, presenting one potential barrier to CKD prevention [23–25]. However, little is known about African Americans’ views on ways to prevent CKD through dietary interventions. Identification of factors likely to influence the success of dietary interventions among African Americans at high risk for CKD could greatly enhance preventive strategies.

We performed a qualitative study to elicit perspectives on CKD prevention via dietary modifications among African Americans of low socioeconomic status (SES), given empirical data suggesting their particularly high risk of adverse kidney outcomes [26–28]. We focused our study on African Americans with a family history of ESRD as this risk factor is more common among African Americans than whites, and is associated with a 2-fold greater risk of developing ESRD [29]. To inform future effectiveness studies aimed at preventing CKD among high-risk African Americans, we examined participants’ views on specific components of the DASH diet which has been associated with lower risk of kidney function decline [11] and other favorable health outcomes [30–33].

## Methods

### Study overview

We performed three focus groups of a total of 17 self-identified African Americans with low socioeconomic status and high risk for CKD to assess their CKD knowledge as well as their perceived barriers and facilitators of CKD prevention through changes in dietary practices. Our approach was guided by the Consolidated Criteria for Reporting Qualitative Research (COREQ) checklist [34].

### Participant selection and setting

African Americans were eligible to participate if they were English-speaking, aged 18-60 years and (1) had a self-reported family history of end-stage renal disease (ESRD, affecting a parent or sibling), (2) had a self-reported biological risk factor for CKD (self-reported history of diabetes, hypertension, cardiovascular disease, HIV or obesity), and (3) were of low self-reported SES (defined as one or more of either less than a high school education, unemployment, lack of health insurance, or annual personal income less than $25,000). We excluded those with a self-report of a CKD diagnosis at the time of enrollment or a history of kidney transplant. We recruited participants through patients treated in dialysis units in the Baltimore, MD metropolitan area. We provided information about our study to dialysis patients, and we asked them to pass this along to their potentially eligible relative(s) who then contacted us directly with interest in participating in the study. All participants provided written consent, and the Institutional Review Board of the Johns Hopkins University approved the study protocol.

### Data collection

We conducted three separate focus groups between June and October 2012. Each participant completed a questionnaire at the beginning of their group session (which was read to them, if needed) assessing socio-demographic information, including age, gender, education, marital status, insurance status, employment status, annual income, and healthcare utilization. The questionnaire also assessed participants’ personal perceptions of their CKD risk by asking “What do you think are your chances of getting kidney failure in the future--low, medium or high?” At the beginning of each focus group, and following the completion of the questionnaire, participants were given and read a one-page flyer titled ‘What is Kidney Disease?’ that provided educational information about the kidney (“The kidneys are small, bean-shaped organs on either side of your spine. The kidneys’ main job is to take out waste and extra fluid from your body. They make urine from this waste”.) [35]; and were provided general details about the treatment of kidney disease in order to provide a framework for the focus group. Additionally, participants were told, “Currently, African Americans develop kidney failure up to 4 times more often than whites, and the two main causes of kidney failure are diabetes (sugar) and high blood pressure”.

All focus group sessions were 90 minutes in length and were facilitated by the same trained moderator, who utilized a standardized guide developed by the study team to conduct the sessions (Table 1 and Additional file 1). We asked participants a series of closed and open-ended questions about their perceptions regarding CKD and its prevention through dietary modifications grouped into three sections (1) knowledge/beliefs, (2) needs and (3) acceptable interventions. At the end of the ‘knowledge/beliefs’ section, we asked “Have you ever heard of the DASH diet (Dietary Approaches to Stop Hypertension diet)? If so, what do you know about it?” We then described the DASH diet as “The DASH diet is rich in fruits, vegetables and low fat dairy foods, and low in salt and saturated fat; and it is a good diet to follow for lowering your chances of getting kidney disease”.

**Table 1:** **Sample questions (10 of a total of 21) from the focus group moderator’s discussion guide**

A. Do you think that what you eat can affect your chances of getting kidney disease? If so, how?	F. The (Dietary Approaches to Stop Hypertension) DASH diet is rich in fruits, vegetables and low fat dairy foods, and low in salt and saturated fat; and it is a good diet to follow for lowering your chances of getting kidney disease. What do you think you would need in order to be able to follow the DASH diet? (e.g. education, money, transportation to better grocery store, etc)
B. What do you think are good foods to eat to lower your chances of getting kidney disease?	G. Do you think you would you be able to find these types of foods in your local food store?
C. What do you think are foods to avoid so that you can lower your chances of getting kidney disease?	H. Do you have everything that you would need in order to prepare your food at home? (e.g. oven, refrigerator, microwave, freezer, electricity/gas)
D. Are there things that your family member with kidney failure did or didn’t eat that you think raised their chances of getting kidney disease?	I. If someone wanted to teach you how to follow the DASH diet, who should that person be?
E. Are there things related to food that you currently do that you think raises your chances of getting kidney disease?	J. Should the person who teaches you about the DASH diet be someone who has followed it before, or does it matter?

### Analysis

The focus group discussions were audio recorded and transcribed verbatim. The grounded theory approach was used for content analysis, given limited available data about the knowledge and beliefs about dietary practices among African Americans at high risk for CKD. Grounded theory is a qualitative tool to derive theories when the literature is lacking on a subject. It uses data sampling through tools such as focus groups to gain understanding of a subject [36, 37]. Two investigators independently reviewed the transcripts after they had been professionally transcribed by an outside company. The coding scheme derived represented concepts addressed by participants during the discussions. These concepts were further refined and categorized to develop a list of key themes regarding perceptions of CKD and potential barriers and facilitators of CKD prevention via dietary changes in this population. The coding process was as follows. Two investigators (T.C. and A.J.) generated their own lists of codes for each individual focus group session. After which, there were constant comparisons within codes and between coders until no new ideas emerged. ATLAS.ti version 6.2.27 (Berlin, Germany) was used for data management and allowed the investigators to create memos and visually depict the emerging themes. A third investigator (D.C.) adjudicated differences in interpretation of emergent themes. Inter-rater reliability occurred with constant comparisons between all coders. Representative excerpts from the participant quotes which corresponded to the emergent themes were selected by the same three investigators for inclusion in the manuscript. Identifiable participant data were stored in a locked file cabinet in the office of the lead investigator and only de-identified data were analyzed by the study team.

## Results

### Participant characteristics

A total of 75 individuals contacted our research team with interest in participating in the study. After initial screening, 20 of 75 met full eligibility criteria, and we invited them to attend a focus group session with a target of 6-8 participants per group. Seventeen individuals ultimately attended one of the three focus groups (consisting of 6, 4, and 7 participants each). No further groups were scheduled due to the emergence of no new major themes in the third session. A majority of participants were female, high school graduates, unmarried, unemployed, and/or earned less than or equal to $10,000 per year. (Table 2) Participants self-reported several medical conditions, including being ≥ 30 pounds overweight (reported by 100%), diabetes, hypertension, heart disease, and HIV. All but one participant had been seen by a clinician in the previous one year. Most (88%) participants perceived their risk of CKD to be ‘low’ or ‘medium’.

**Table 2:** **Focus group participant characteristics**

Characteristic	n = 17 (%)
Age in years, mean (SD)	39.8 (12.4)
Female gender	10 (59)
Highest level of education	
High school	10 (59)
2 year college	2 (12)
College or graduate/professional school	5 (30)
Marital status	
Married	4 (24)
Living with a partner	2 (12)
Separated	2 (12)
Never married	9 (53)
Health insurance	
Private	2 (12)
Medicare or Medicaid	8 (47)
None	5 (29)
Other	2 (12)
Employment	
Unemployed	11 (65)
Part time	3 (18)
Full time	3 (18)
Yearly income ≤ $10,000	14 (83)
Chronic conditions	
Obesity (≥ 30 lbs overweight)	17 (100)
Diabetes	4 (24)
Hypertension	9 (53)
Heart disease	2 (12)
HIV	1 (6)
Family member with CKD*	
Father	6 (35)
Mother	3 (18)
Sibling(s)	10 (59)
Other	1 (6)
Time since last seen by a clinician	
Less than 3 months	11 (65)
Less than a year	5 (29)
More than a year	1 (6)

*Not mutually exclusive.

### Knowledge about CKD causes and risk factors

Participants expressed several ideas about potential causes of CKD and CKD risk factors, encompassed by four main themes (Table 3):

**Table 3:** **Emergent themes**

Knowledge about CKD causes and risk factors	Perceived barriers to CKD prevention through dietary changes	Acceptable interventions and facilitators of CKD prevention through dietary changes
1 CKD can be caused by medications or inadequate water intake	1 Healthy foods are expensive and unavailable in certain neighborhoods	1 Education and nutritional counseling
2 CKD can be caused by frequent consumption of certain foods	2 Unhealthy foods are more convenient to prepare or access	2 Raising CKD awareness
3 CKD is often caused by other chronic diseases	3 Unhealthy dietary practices have been a lifelong habit	3 Home-based and family Interventions to increase DASH diet adherence
4 Family history and ethnicity are risk factors for CKD	4 Family members’ dietary requirements and preferences challenge ability to change habits	4 Community-based interventions

#### CKD can be caused by medications or inadequate water intake

Participants believed that kidney problems could be caused by or exacerbated by medication use, and some believed dietary supplements might help to prevent kidney disease. Participants also believed that inadequate water intake could lead to CKD, as could the frequent consumption of certain other beverages (e.g. soft drinks and alcoholic beverages).

One participant described some thoughts about medications and kidney injury:

*“In his case [referring to a relative with kidney disease], it was more the fact that the medication that he was taking was overpowering his kidneys. So by overpowering his kidneys, his kidneys couldn’t flush them out. And the more he was taking the medicine, the more he couldn’t flush them out in time enough. So it broke them down”.*

#### CKD can be caused by frequent consumption of certain foods

Participants commonly reported their beliefs about the in a relation between consumption of ‘unhealthy’ foods and increased risk of CKD. Participants also reported beliefs that avoidance of fast, fried, canned or processed foods could potentially lower an individual’s risk of CKD. They also believed that food preparation (including under- or overcooking food, and the use of seasonings) could play a role in determining CKD risk. A participant commented:

*“I do think I'm at high risk ‘cause I don't really watch what I eat. I eat a lot of fried foods like she stated, starches, things like that. I really am at high risk. I've tried to slow down”.*

Another participant commented:

*“I think most people lack more vegetables than anything. Even though sometimes a lot of people think some vegetables may be good for you, but it's what we put on the vegetables as well that doesn't help our diets as well…”*

#### CKD is often caused by other chronic diseases

While some participants were unaware that kidney disease can be caused by other conditions, many associated kidney disease with chronic diseases such as hypertension and diabetes. They also identified that dietary habits may relate to risk of CKD through its influence on risk of these other chronic conditions. For instance, one participant commented:

*“Because my father got it through diabetes, and it's the food that he ate which caused him to obtain diabetes, and then he just went downhill from there. And I had actually had gestational with my daughter, and I had to change my whole diet around. So I believe that that's a big reason why”.*

#### Family history and ethnicity are risk factors for CKD

Participants believed family history plays a role in an individual’s risk of CKD, and they shared many insights into their family members’ experiences with CKD.

*“I got a niece who just had a kidney transplant, and I got an older brother, he's on dialysis, right, so I got some fears that I might get it one day, you know what I mean? I'm not saying a real high risk, but a medium risk, you know, see what I'm saying? Because I've got two family members suffering with, you know, my mother never had it, but, you know, I think it runs in my family”.*

Participants also shared their perceptions of racial differences in kidney disease burden. One participant commented:

*“I still have that problem of, why is there so many African Americans? And I do know that there are a lot of Caucasians that end up suffering from dialysis, but it seems like they don't suffer until they get like their eighties, like between 75, they're 80, where so our community is so young”.*

### Perceived barriers to CKD prevention through dietary changes

No participants reported prior knowledge of the DASH diet when asked by our moderator. Following a description of the DASH diet, focus group participants described factors that might hinder their ability to follow such a dietary pattern and potentially lower their risk of CKD. These factors were encompassed by four main themes (Table 3):

#### Healthy foods are expensive and unavailable in certain neighborhoods

Several group members reported that financial limitations pose a barrier to following a healthful diet and that fresh produce is often not plentiful in low-income neighborhoods.

One participant stated:

*“This is somewhat off the beaten path, but if your socioeconomic conditions don’t dictate that you have the money, then that puts you at a disadvantage to someone that has the wherewithal to buy these fruits and vegetables, so therefore, you’re in a catch-22 to a certain extent or maybe you have to push something else aside to do that. Change your budget around a little bit, you know?”*

Another participant commented:

*“And what we have in our neighborhoods, and most low-income neighborhoods, is fast-food restaurants everywhere. You hardly see a farmers' market or fresh produce stand, or even fresh produce in the supermarket. And as soon as you walk in the market the first thing you see is cakes, cookies, chips, cereal with loads of sugar”.*

#### Unhealthy foods are more convenient to prepare or access

Several participants reported convenience of food preparation as influencing dietary choices and a potential barrier to healthy eating. One participant commented:

*“Yes, the fried foods, the starches are a lot easier to cook. It's not easier to cook, it's quicker. It's a quick meal, 30 minutes and I'm done. But I need to learn how to incorporate some green vegetables and salads in there as well”.*

#### Unhealthy dietary practices have been a lifelong habit

Participants commonly reported that unhealthy dietary habits began during childhood and extended into their adulthoods. They shared their perceptions regarding the difficulty of changing long-standing lifestyle patterns. One participant remarked:

*“It’s generational, how we was brought up”.*

#### Family members’ dietary requirements and preferences challenge ability to change habits

While some participants reported that they lived alone, most reported living with family members and/or friends. Those who lived with others expressed their dietary practices were affected by the dietary requirements and preferences of household members. One participant commented:

*“Yeah, because my family- all of them eat fried foods and, you know, unhealthy foods. Order out pizza and all of that”.*

### Acceptable interventions and facilitators of CKD prevention through dietary changes

Participants shared their thoughts on interventions that would be acceptable for enhancing their ability to follow a DASH-like diet and shared ideas for raising awareness about CKD prevention, encompassed by 5 main areas (Table 3):

#### Education and nutritional counseling

Participants reported they believed the ideal person(s) to educate people about recommended dietary changes included doctors, nutritionists/dieticians and social workers --though some felt the term “social worker” held a negative societal connotation. While participants reported they did not feel it mattered whether educators were members of their community, some felt that in order to teach a certain dietary pattern, the educator would have to follow it as well.

*“Because if you’re going try to teach me something, you can’t tell me nothing that you don’t know. So you have to lead by example, so I’m going to have to know that you tasted it, tried it, and seen for yourself that it works”.*

Participants felt that once educated, the onus of behavior change was on the individual. For example, some had met with nutritionists in the past but related they struggled to adhere to the recommended diet thereafter. One participant explained,

*“I have met with [a nutritionist], but I go off. I just go off. I'd be good for two or three weeks, and then I want to give myself a treat, and once I do that, I'm off again”.*

#### Raising CKD Awareness

Participants conveyed their support for efforts to raise community awareness of CKD. A participant stated,

*“I think there should be more informative sessions about the kidney, chronic kidney disease because also people, especially black Americans, are not aware of the food that they eat that affects these things. So that's probably one way of trying to reduce it”.*

#### Home-based and Family Interventions to Increase DASH Diet Adherence

Participants identified that support in the home would facilitate healthy lifestyle changes, including assistance with meal preparation. When asked by our moderator, “Do you have everything that you would need in order to prepare your food at home?”, all participants acknowledged that they had adequate appliances in the home to support meal preparation. While some felt it would be a challenge to engage family members in following the DASH diet, others described ways in which following the DASH diet would be feasible for their families:

*“I think one of the things that may help you with the DASH diet is if you sit down with other family members and you discuss the situation about it trying to be from a preventive standpoint, say we’re all going to go on it a support-type thing, and say let’s try it this way”.*

There was also some discussion about whether, as part of an intervention, participants would allow others into their homes to assess whether they had DASH-diet adherent foods. Most participants felt this would be acceptable. One person remarked:

*“I say yes because the whole purpose of them coming there is because you’ve accepted the fact that you need to change your eating habits. So they’re bringing to the forefront what is considered to be bad food that’s damaging to your system or what’s damaging to hurt you. So you invite them into the presence of your home, you invite them into your kitchen, this is where the problem lies. Why not? Why not open your cabinets? Remove the sheets. Remove the veil. Let it out”.*

#### Community-based interventions

Participants offered several ideas for acceptable community-based interventions including DASH diet gatherings in the community, teaching children about healthful eating at schools, church based activities and support groups. One participant described how a group intervention held in the home would help to establish a supportive network:

*“Or you could even have like a DASH diet party. You know, have a Tupperware party? Just have a DASH diet party… That way you can do it in a group setting at your home so you won't feel uncomfortable”.*

Participants expressed concern for the future of children growing up without healthful dietary practices and described potential school-based interventions. For example,

*“I think hitting the elementary school level and trying to motivate them because again those are going to be the people that grow up to have these bad eating habits or just be ignorant to some of the issues that can cause kidney failure”.*

#### Food vouchers and other financial interventions

Some participants endorsed financial interventions as a means to address low adherence to healthful diets among low income individuals. One participant remarked:

*“I think it should be a program like that for different people with terminal illnesses or illnesses like kidney disease, because some people may not be able to afford to go get fresh vegetables and things, and if they had the vouchers to go get the fruits and vegetables or whatever they might need, they might help them in their diet”.*

## Discussion

In this qualitative study, African Americans of low SES and who are at high risk for developing CKD perceived several barriers to CKD prevention through dietary change, including the expense and unavailability of healthy foods, family member preferences, convenience of unhealthy foods, and inability to break lifelong habits. They identified vouchers for healthy foods, home and/or family-based interventions, nutritional counseling and group gatherings for persons interested in making dietary changes as potentially acceptable approaches to preventing CKD.

As in a previous study of low-income, hypertensive African Americans’ views of the DASH diet [38], our participants identified both socioeconomic and cultural barriers to following a DASH-like diet--and the two may be interrelated. A key barrier identified by our participants was limited accessibility and high cost of healthful foods. Consistent with participants’ perceptions, the availability of affordable and nutritious food has been shown to be limited in many low income and/or minority communities, including those in Baltimore City [39, 40]. Additionally, a recent meta-analysis of studies spanning 10 countries reported that healthier foods/diet patterns are more costly than less healthy foods [41]. Notably, these barriers to dietary modifications have been overcome in some low income communities who have implemented programs to increase availability of healthful and affordable foods [42, 43]. However, improving access alone may not increase consumption of healthful foods if they are perceived to be culturally inappropriate [38] or less palatable [44].

Family member food preferences posed a significant challenge to our participants, and likely relate, in part, to cultural norms among African Americans [38]. There is a perception among some African Americans that healthful eating requires giving up part of their cultural heritage and conforming to the dominant culture [45]. The idea of giving up cultural norms is particularly at odds with the altruistic concept of family-based collectivism, which places the interests and needs of the family before those of the individual and is a core value for many racial minority groups [46]. While DASH diet recommendations have been presented in the context of soul food and other traditional diets in the African American community [47, 48], effective adaptions of these recommendations have not fully penetrated high-risk populations. Thus, further efforts to make DASH diet recommendations which are culturally-tailored and acceptable to persons at risk for CKD and other consequences of hypertension, are warranted. Such approaches may aid in breaking the life-long habits our participants identified as significant barriers to dietary changes.

Potential facilitators of CKD prevention through dietary modifications detailed by our participants included individual, as well as home and community-based programs and interventions. Receipt of education from an individual who had personal experience with making dietary changes was viewed favorably by our participants. Similarly, focus group participants in an obesity prevention study among African American women preferred to learn about diet and physical activity from someone like them, as opposed to, for example, celebrities who were not viewed as credible given their access to resources unavailable to most [49].

The notable finding that none of our participants had prior knowledge of the DASH diet (despite over half reporting a diagnosis of hypertension) suggests substantial gaps in dissemination of evidence-based dietary approaches exist in this population, which is an opportunity for clinicians caring for African Americans at high risk for CKD. Home-based educational programs, as suggested by our participants, may be particularly effective in reaching disadvantaged populations who may face transportation, childcare and other barriers to clinic-based services [50]. For example, a home-based diabetes education trial among Hispanics led to significant reduction in hemoglobin A1c levels [51].

At age 40 years (the mean age of our study participants), African American men and women in the general U.S. population have a 16.8% and 18.9%, respectively, lifetime risk of developing advanced CKD (estimated glomerular filtration rate <30 ml/min/1.73 m^2^) [52]. While participants in our study reported some knowledge of established causes and risk factors for CKD, and all had at least one biological risk factor for kidney disease (i.e. diabetes or hypertension), many did not consider themselves to be at high risk of developing CKD. For example, participants were generally aware that family history is an important determinant of CKD [53, 54] and all participants had at least one first degree family member with ESRD. Yet, consistent with a prior study from our group which was largely composed of African Americans at high-risk for CKD [23], few participants perceived their personal risk of CKD to be high. While this finding argues for increased education among African Americans at high risk for CKD, risk awareness alone may not suffice. Perceived susceptibility to CKD has been associated with poorer blood pressure management [23], suggesting that fatalistic attitudes about CKD might impede its prevention, perhaps particularly when patients face significant barriers to managing risk factors.

Our study had limitations which should be considered. First, our focus groups included a relatively small number of participants and were conducted in an urban U.S. city. Additional concepts or themes might be generated by interviewing additional participants in the study area or participants in other settings. Furthermore, participants of research studies may be more activated around their health than non-participants, which could have influenced group discussions. Second, we used self-reported data to determine participants’ clinical status, and did not recruit participants across strata of socio-demographic or clinical factors, therefore, certain groups may have been underrepresented. Third, we included participants with at least one indicator of low SES, however, individual differences across indicators (e.g. high educational attainment but low annual income) may have influenced participant responses. Fourth, we did not return the transcripts to participants to review, which may have impaired the accuracy of the inferences made from participant statements. Fifth, we did not assess the health literacy of our participants, and we did not specifically delineate between objective and perceived knowledge. By asking open-ended questions and asking participants to elaborate, we facilitated discussion of what was most likely perceived knowledge. To better differentiate, future work might include a validated survey of a similar population. Lastly, while the DASH diet has been associated with many favorable health outcomes, it has not been evaluated for its potential effect on CKD prevention in controlled studies. Thus, our study may serve to generate hypotheses for such future interventions.

To our knowledge, our study is among the first to report detailed views about the role of dietary interventions in CKD prevention among African Americans at high risk for CKD. We extended prior studies of barriers to healthful eating among African Americans by also elucidating potential facilitators of change in a population in need of targeted interventions As the body of literature on effective dietary strategies for reducing kidney injury continues to grow [13, 14] our findings may inform translation of these strategies to vulnerable populations.

## Conclusions

African Americans of low SES and who are at high risk for developing CKD demonstrated limited perception of their risk status, but identified multiple barriers and potential facilitators of CKD prevention via dietary modifications which can inform tailored interventions and public health initiatives.

## Electronic supplementary material

Additional file 1: **Focus group moderator’s guide.** (DOCX 38 KB)
