# Supplementary material for: Perceived barriers and facilitators of using dietary modification for CKD prevention among African Americans of low socioeconomic status: a qualitative study
Source: BMC Nephrol. 2014 Dec 6;15:194. doi: 10.1186/1471-2369-15-194 (PMC4268853; doi:10.1186/1471-2369-15-194)
Supplement: Supplementary file 1 — Additional file 1: Focus group moderator’s guide. (DOCX 38 KB) [file 12882_2014_881_MOESM1_ESM.docx]

FOCUS GROUP

MODERATOR’S GUIDE

**PART 1. Introduction**

**A. Greetings**

Greet the participants warmly and similarly, with same words.

Example: “I’m glad you came today; thank you for coming”

Pick a seat for yourself where you can face everyone – for example the head of the table – without turning around.

Thank each by name and introduce yourself:

“My name is ________, and I will be your moderator today”

| **B. The Purpose [5 minutes]**  After everyone is seated and ready to begin:   - Thank you again for coming. - This research study is focused on kidney disease, and you are each family members of someone with kidney failure - **Read 1 page flyer about the kidneys to the group - Currently, African Americans develop kidney failure up to 4 times more often than whites, and the 2 main causes of kidney failure are diabetes (sugar) and high blood pressure - Our main purpose is to reduce this difference by finding ways to prevent people with family members who have kidney disease from also developing the disease. - Our discussion will be focused around the types of **food** that you eat - We will use the information you provide to us during this session to develop our prevention program.   As you heard when you agreed to participate, our discussion will be tape recorded. Our research coordinator is going to turn on the tape recorder now. We use tape recorders so we can recall all the information you have shared with us. When the tapes are transcribed, you will be identified by a number only. The transcriptionist is off site, so your identity will be confidential. Does anyone have any questions? | **6:15pm** |
| --- | --- |
| **C. Introduction of Participants and Rules [10 min]**  We deeply appreciate your coming today. Every opinion is important, and I’m looking forward to hearing from each of you. **We have just a couple of rules**:  1) Everyone participates  2) Every opinion is important – **we want to hear from everyone, I may have to cut you off to give others a chance to share.**  3) Our meeting is confidential – between ourselves. **What is said here, stays here.**  4) The tape recorder needs help - try to speak clearly.  5) All cell phones or pagers off  We’ll be talking together for about an hour and a half. Feel free to get some refreshments quietly during the conversation.  Introductions  Let’s start with a brief introduction from each of you. Please state ***“I am Number XX”*** when you first speak. This is for the transcriptionist, so he or she will be able to know the sound of your voice, in case we want to link your comments. [Go around the table …] | **6:20 pm** |
| **PART 2. Discussion**  Okay that’s great. Let’s get started with our discussion.  **Knowledge/Beliefs**   1. Do you think that what you eat can affect your chances of getting kidney disease? If so, how? 2. What do you think are good foods to eat to lower your chances of getting kidney disease? 3. What do you think are foods to avoid so that you can lower your chances of getting kidney disease? 4. Are there things that your family member with kidney failure did or didn’t eat that you think raised their chances of getting kidney disease? 5. Are there things related to food that you currently do that you think raises your chances of getting kidney disease? 6. Have you ever heard of the DASH diet (Dietary Approaches to Stop Hypertension diet)? If so, what do you know about it? | **6:30 pm** |
| **Needs**  The DASH diet is rich in fruits, vegetables and low fat dairy foods, and low in salt and saturated fat; and it is a good diet to follow for lowering your chances of getting kidney disease   1. What do you think you would need in order to be able to follow the DASH diet? *(e.g. education, money, transportation to better grocery store, etc)* 2. Do you think you would you be able to find these types of foods in your local food store? 3. How often do you shop at grocery stores that sell these types of foods? 4. Do you cook or eat out most days of the week? 5. Do you have everything that you would need in order to prepare your food at home? (e.g. oven, refrigerator, microwave, freezer, electricity/gas) 6. Do you think the other members of your family would join you in following the DASH diet? Why, or Why Not? | **7:00 pm** |
| **Acceptable Interventions**     1. If someone wanted to teach you how to follow the DASH diet, who should that person be? 2. Should the person who teaches you about the DASH diet be someone who has followed it before, or does it matter? 3. Should it be someone from your neighborhood/community? 4. How should the education about the DASH diet be provided? (e.g. **face-to-face, via text message, phone conversation, website, group seminar**) 5. How would you feel about someone coming to your home to teach you about the DASH diet? 6. Would you be ok with someone looking in your kitchen cabinets and refrigerator to see what types of food are currently there? 7. Would a meeting with a social worker be useful to you, in helping you learn how you might be able to follow the DASH diet? 8. Has anyone ever met with a nutritionist? Do you think that would be useful? | **7:30 pm** |
| **8. Anything Else:** What else is important that I haven’t asked about? **[10 minutes]**  Probe:   1. Is there anything else you would like to share? | **7:40 pm** |

**PART 3. Conclusion**

For each group:

We are going to turn off the tape recorder now and then we’ll have a few minutes for any other comments you want to give to me.

Thank the participants for coming.

Our principal investigator will be handing out the payment. Please see Dr. Crews and she will have you sign a form so you can get your payment.

**Part 4. Helpful Tips on Moderating Focus Groups**

1) Examples of probing questions:

“Tell us more about that.”

“Would you explain further?” “Can you give me an example?”

“Is there any thing else?”

“Please describe what you mean.”

“I don’t understand.”

2) Limit your comments to:

For ending -- “Thanks for those comments”

To continue -- “I see” or “Tell us more”

To change to other person -- “We’d like your opinion now”

Adjust your tone to demonstrate sympathy and understanding.

3) Ask different participants in different order, not around the table every time.

4) Listen closely – if you don’t understand, ask for clarification right away. Feel free to stop response by saying, “Just so we can understand, you’re saying… and re-state the participant’s words,” or “Can you re-state that?”

5) For the first half hour, have each person state his/her number when answering.

6) Everybody answers each question: Ask each participant for an answer to each question.

7) Be sure that each participant has an opportunity to comment in all 3 domains.

8) Make sure questions are phrased in a way that people can express different experiences and responses.

9) Be prepared to shift gears quickly, E.g. if the discussion naturally leads to a different question, the question order can be altered in order to follow the natural flow of conversation.

**Part 5. Additional notes:**

1. Introductions:

Greet all participants similarly, with a handshake and a warm brief greeting, e.g., “I‘m ----, the moderator for this evening and I’m so glad you could be a part of our group.”

2. Be clear with instructions, e.g.,

“The bathroom is out the door, to the left, at the end of the hall marked ‘ladies’”

“Please give us a brief introduction of 2-3 sentences”

3. For participants who go on too long

“Thank you for all that information; that’s enough for now. We may get back to that issue later in our conversation”

4. For ‘sidebar’ conversations (participants talking together off group theme)

“Please tell us all about what you’re talking about; we’d all like to know.”

“That’s not one of our assigned topics this evening – we’ll have time to talk together at the end of the session”

5. For personal conversations or comments not related to themes

“We’ll have some time at the end of the session to talk together informally, and we can get back to that issue”

6. What to do if…

a) Someone starts crying?

"Health is always an emotional issue. Do you need to take some time out?"

b) No one says something?

"This is a complex and emotional issue. I think some-one here has a story they can tell us."

c) You finish too early?

1. Look back over the questions and think if there is one or another you did not hear much on.

2. Ask the group: "What other related topics have we missed here?"
